# A method to concatenate multiple short time series for evaluating dynamic behaviour during walking

**DOI:** 10.1371/journal.pone.0218594

**Published:** 2019-06-21

**Authors:** Stefan Orter, Deepak K. Ravi, Navrag B. Singh, Florian Vogl, William R. Taylor, Niklas König Ignasiak

**Affiliations:** 1 Institute for Biomechanics, ETH Zürich, Zurich, Switzerland; 2 Department of Physical Therapy, Chapman University, Irvine, California, United States of America; Berner Fachhochschule, SWITZERLAND

## Abstract

Gait variability is a sensitive metric for assessing functional deficits in individuals with mobility impairments. To correctly represent the temporal evolution of gait kinematics, nonlinear measures require extended and uninterrupted time series. In this study, we present and validate a novel algorithm for concatenating multiple time-series in order to allow the nonlinear analysis of gait data from standard and unrestricted overground walking protocols. The full-body gait patterns of twenty healthy subjects were captured during five walking trials (at least 5 minutes) on a treadmill under different weight perturbation conditions. The collected time series were cut into multiple shorter time series of varying lengths and subsequently concatenated using a novel algorithm that identifies similar poses in successive time series in order to determine an optimal concatenation time point. After alignment of the datasets, the approach then concatenated the data to provide a smooth transition. Nonlinear measures to assess stability (Largest Lyapunov Exponent, LyE) and regularity (Sample Entropy, SE) were calculated in order to quantify the efficacy of the concatenation approach using intra-class correlation coefficients, standard error of measurement and paired effect sizes. Our results indicate overall good agreement between the full uninterrupted and the concatenated time series for LyE. However, SE was more sensitive to the proposed concatenation algorithm and might lead to false interpretation of physiological gait signals. This approach opens perspectives for analysis of dynamic stability of gait data from physiological overground walking protocols, but also the re-processing and estimation of nonlinear metrics from previously collected datasets.

## Introduction

Walking continuously is instrumental for humans to perform various activities of daily living and lead an independent life. In order to walk continuously, complex mechanisms within the human sensory motor system provide the necessary timing, coordination and balance such that the interplay between the centre of mass and the base of support are regulated in a repetitive manner [1]. Inter-cycle variations during continuous walking, commonly termed gait variability, are therefore an indicator of how repetitive walking is governed by the neuro-muscular system [2, 3]. As such, gait variability quantifies an individual’s ability to adapt to natural internal (e.g. noise in the human neuromuscular system [4]), as well as external perturbations during walking and thus prevent falling [5, 6]. Gait variability is therefore the result of a continuous dynamic system recovering from perturbations. Parameters such as the largest Lyapunov exponent or sample entropy have previously been used to assess this dynamic behaviour, in order to assess dynamic stability and regularity of human locomotion [7–9]. Unlike linear metrics of gait variability, these nonlinear parameters derived from gait-time series assume that consecutive gait cycles are dependent upon one-another [2]. This assumption is essential in assessing motor adaptation strategies during walking and therefore nonlinear parameters have a clear benefit over linear metrics for characterising gait variability. However, in order to ensure validity of these measures, it is also critical that gait patterns are recorded in a consecutive manner [10], as well as that the derived time series is not interrupted [11].

One obstacle when investigating the dynamic nature of walking in laboratory settings is the limited field of view of optical motion capture systems, consequently constraining the collection of extended time series. Motorized treadmills are generally used to overcome this issue, but due to their prescribed walking speeds, non-physiological walking patterns occur [12–14]. In order to collect consecutive cycles of overground walking, a novel protocol—the so-called 8-walk—was recently proposed, where subjects are requested to follow a pattern of eight walking track in order to record multiple non-interrupted (i.e. continuous) bouts of walking [10]. While this protocol has been shown to reliably estimate linear measures of gait variability and allows the collection of physiological walking patterns, the recorded time-series often become discontinuous when participants walk out of the motion capture field of view during turning, thereby violating the requirement for non-interrupted time series recordings when evaluating dynamic gait stability [10].

Our goal is to allow researchers to collect data using standard and unrestricted overground walking protocols, while still profiting from the advantages of nonlinear analysis methods. This paper therefore describes a novel method to concatenate such discontinuous sets of time-series, in order to yield a single large time-series, which then adheres to the requirements of nonlinear methods for evaluating the dynamic nature of walking. Importantly, we aim to develop a generic method that allows non-linear analysis of full body kinematics from motion capture data, independent of the walking protocol (overground vs. treadmill), as well as the type of trajectory (position, velocity or angular kinematics) in order to be applicable in a variety of research settings. Furthermore, validation of the concatenation approach on both normal and perturbed walking will allow previously collected gait data to be re-analysed using nonlinear algorithms. Results quantifying the efficacy of our concatenation method are presented based on two widely used nonlinear measures to assess stability (Largest Lyapunov Exponent—LyE) and regularity (Sample Entropy—SE) [11].

## Methods

Twenty healthy subjects (10 males and 10 females) provided written informed consent prior to the beginning of experimental procedures, which were approved by the ETH Zurich ethics committee. Their mean (SD) age, body height and mass were 27.0 (4.2) years, 175.7 (8.9) cm, and 71.6 (10.9) kg respectively. None of the subjects had any form of acute musculoskeletal or neurological disorders or pain that may have affected their ability to walk.

Kinematic data was collected using an optical motion-capture system (Vicon Motion Systems Ltd. UK) using 10 Vicon cameras (T160) at a sampling frequency of 100Hz. A whole-body marker set consisting of 62 retro-reflective markers was used. While dynamic behaviour of walking was evaluated from the heel marker of the right foot, all remaining markers were used only in order to identify similar poses during the concatenation process. In order to fulfil the continuity requirements for nonlinear measures in the development of the concatenation algorithm, the experimental procedure consisted of three sets of 5-minute barefoot walking captured on a treadmill at self-selected speeds (0.96 ± 0.10 m/s). In order to draw conclusions on the effectiveness of the concatenation procedure in assessing the dynamic behaviour of walking under the effects of perturbation, participants also performed treadmill walking trials with +40% bodyweight (*wei40*) and -40% bodyweight (*har40*), captured in randomized order. For trials with addition of external weight, a weight vest was worn, whereas trials with bodyweight support were performed using a clinical harness system.

### Data pre-processing

For each trial, the recorded 5-minute full time series (*fullTS*) were separated with the intention to simulate three different sets of shorter time series. In order to create single time series with the same number of gait cycles (*C*_*in*_) within each set of short times series, heel strikes (HSs) were identified using the stable foot velocity algorithm for defining gait cycles [15], where a complete gait cycle was defined as the time between a HS and the following consecutive HS from the right foot. After *C*_*in*_ cycles, a number of cycles (*C*_*ex*_) were excluded (i.e. removed), followed by the inclusion of a further *C*_*in*_ cycles, and this procedure was repeated until the end of the *fullTS*. In this manner, three short time series were created for each trial using *C*_*in*_/*C*_*ex*_ = 10 / 1 (*cut1001*), 8 / 3 (*cut0803*), and 6 / 5 (*cut0605*).

### Concatenation

**Fig 1:**
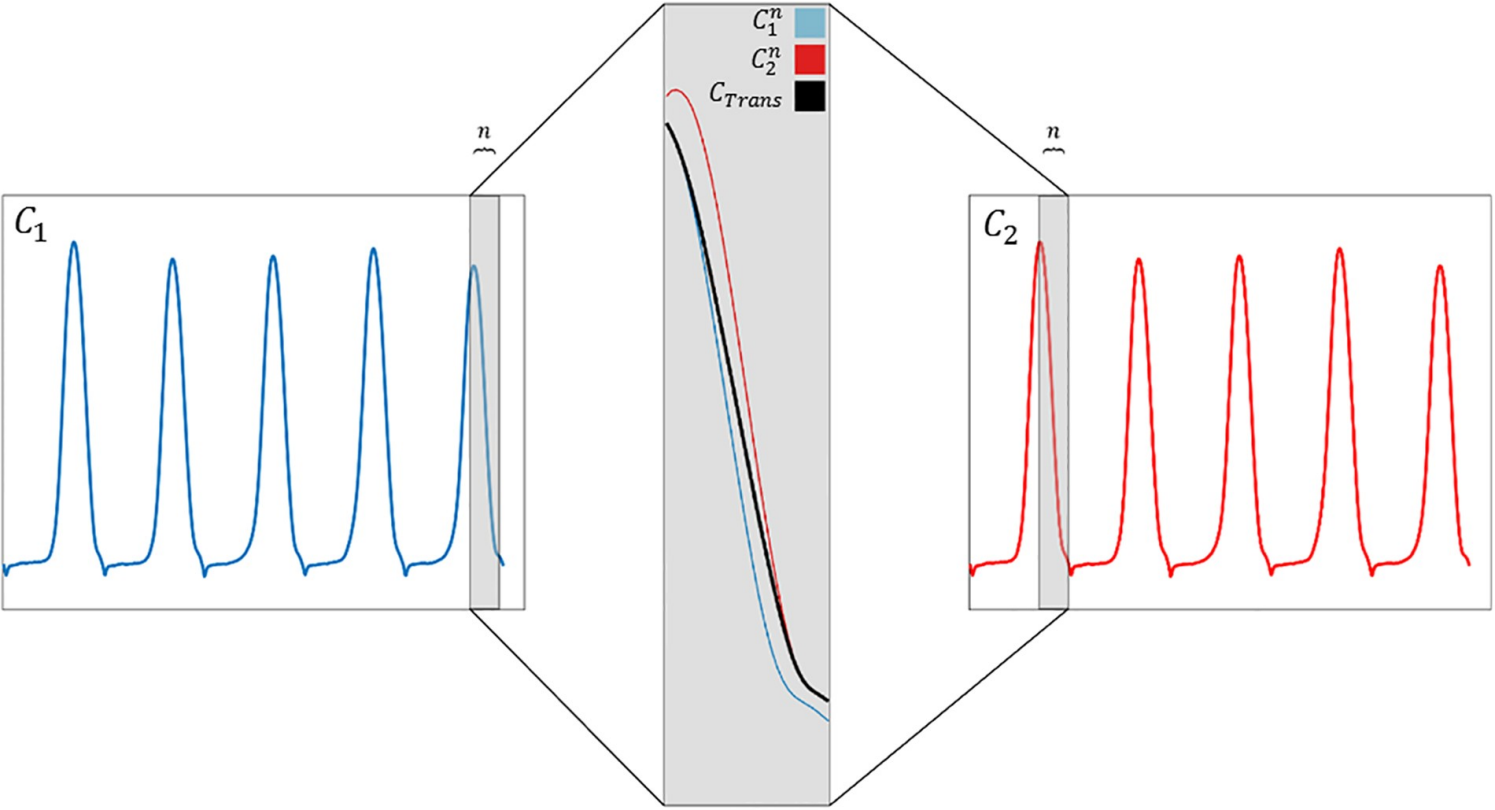
Demonstration of a typical transition between two consecutive short times series (Eq 2). The grey shaded area highlights the selected transition period (as determined by the first local minima of the distance function), with the concatenation result (*C*_*Trans*_) shown in black.

In order to concatenate the pre-processed short time series, an algorithm that is based on pose identification and matching was used [16], in which the best possible match in terms of kinematic pose between the final frames of a *C*_*in*_ time series and the early frames of the following *C*_*in*_ time series was initially identified. This strategy was chosen to ensure that two similar heel positions would be concatenated and thus to avoid adding artifacts in the interpolated trajectories. These kinematic poses were then transformed in order to obtain smooth transitions between the each consecutive *C*_*in*_ series [16]. The concatenation procedure was thus performed as follows:

- Extract $C_{1}^{n}$ as the last *n* = 33 frames of *C*_1_, over which the transition from *C*_1_ and *C*_2_ should be achieved, where ′*n*′ has been chosen corresponding to a window of about a third of the sampling frequency [17]
- Extract $C_{2}^{n,v}$ as the same number *n* of frames from *C*_2_ starting from frame index *v*. Repeat this procedure for all 0 ≤ *v* ≤ *N* − *n* + 1, where *N* is the total number of frames in *C*_2_.
- Calculate the squared distance
  $$D(C_{1}^{n},C_{2}^{n,v})=\sum_{m=0}^{M-1}\sum_{j=0}^{n-1}{w_{m}∥p_{m,j}-T_{j}^{v}p_{m,j}^{\prime }∥}^{2}$$ (1)
  where *p*_*m*,*j*_ is the position of marker *m* for frame *j* of $C_{1}^{n}$ and $p_{m,j}^{\prime }$ is the position of the corresponding marker and corresponding frame in $C_{2}^{n,v}$. *w*_*m*_ further represents the weighting assigned to each marker 0 ≤ *m* ≤ *M*– 1, with *M* the total number of markers, and $T_{j}^{v}$ is an optimized linear transformation from $C_{2,j}^{n,v}$ to $C_{1,j}^{n}$, comprised of a rotation around the vertical axis and a translation in the horizontal plane (which was particularly chosen with the idea that we are able to align the data orientation resulting from the 8-walk protocol). The closed form solution of the transformation $T_{j}^{v}$ is presented in the literature [16].
- In this formulation, weights 0 ≤ *w*_*m*_ ≤ 1 were assigned to each marker, where high weightings were assigned to bony landmarks (over soft tissue markers) and those in proximity to the heel marker.
- The local minima of the function $D(C_{1}^{n},C_{2}^{n,v})$ with respect to *v* therefore allows identification of the frames in *C*_2_ in which the poses are most similar. For this study, the first local minimum $v=V\;\text{of}\;D(C_{1}^{n},C_{2}^{n,v})$ was chosen, meaning that the concatenation of *C*_1_ to *C*_2_ started at the beginning of $C_{1}^{n}$ and transitioned over *n* frames into $C_{2}^{n,v=V}$, at which point the remainder of *C*_2_ followed.
- In order to ensure a smooth spatial transition from *C*_1_ to *C*_2_, the marker positions of each frame index 0 ≤ *j* ≤ *n* − 1 within the two selected concatenation windows $C_{1}^{n},C_{2}^{n,V}$ were linearly interpolated (Fig 1) as follows:
  $$C_{Trans}=\alpha (j)C_{1,j}^{n}+[1-\alpha (j)]C_{2,j}^{n,V}$$ (2)
  where the interpolation coefficient *α*(*j*) is given by:
  $$\alpha (j)=2{\left(\frac{j+1}{n}\right)}^{3}-3{\left(\frac{j+1}{n}\right)}^{2}+1$$ (3)
  [16]
- With the concatenation of *C*_1_ and *C*_2_ complete, this procedure is repeated with *C*_2_ as the new *C*_1_ to be concatenated to the next consecutive *C*_*in*_ series.

In order to impartially test the validity of the concatenation process for assessing nonlinear properties of gait, each concatenated time series (*cut1001*, *cut0803 or cut0605)* was cropped to an equal length (of the shortest time series), but which included a different number of concatenations. Data was not filtered in order to avoid unintentional manipulation of nonlinear stride dynamics [18].

### Nonlinear analyses

The raw vertical position of the right heel marker was taken as the input data for all nonlinear analyses. *Lyapunov exponent*: As a measure for the stability of a time series, the largest Lyapunov exponent, LyE (τ, dim), was calculated according to Wolf [19]. The embedding dimensions (dim) and the time lag (τ) were determined separately for each trial, using a false-nearest-neighbours approach (FNN) and average mutual information (AMI) method respectively [20, 21]. The Wolf algorithm calculates the average rate of divergence of two neighbouring data trajectories in the state space. Here, trajectory divergence was observed over 3 consecutive points, with a minimal (i.e. noise floor) and maximal attractor probe length of 10^−4^ and a tenth of the input data range, respectively.

*Sample entropy*: In order to quantify regularity in the data, a modified sample entropy measure, as suggested by Govindan and colleagues, that considers time delay and dimension of the time series, SE (τ, dim, r), was used. While τ and dim were obtained through FNN and AMI approach, r is chosen as 0.2 times standard deviation of the data trial, for all trials [22–25].

**Fig 2:**
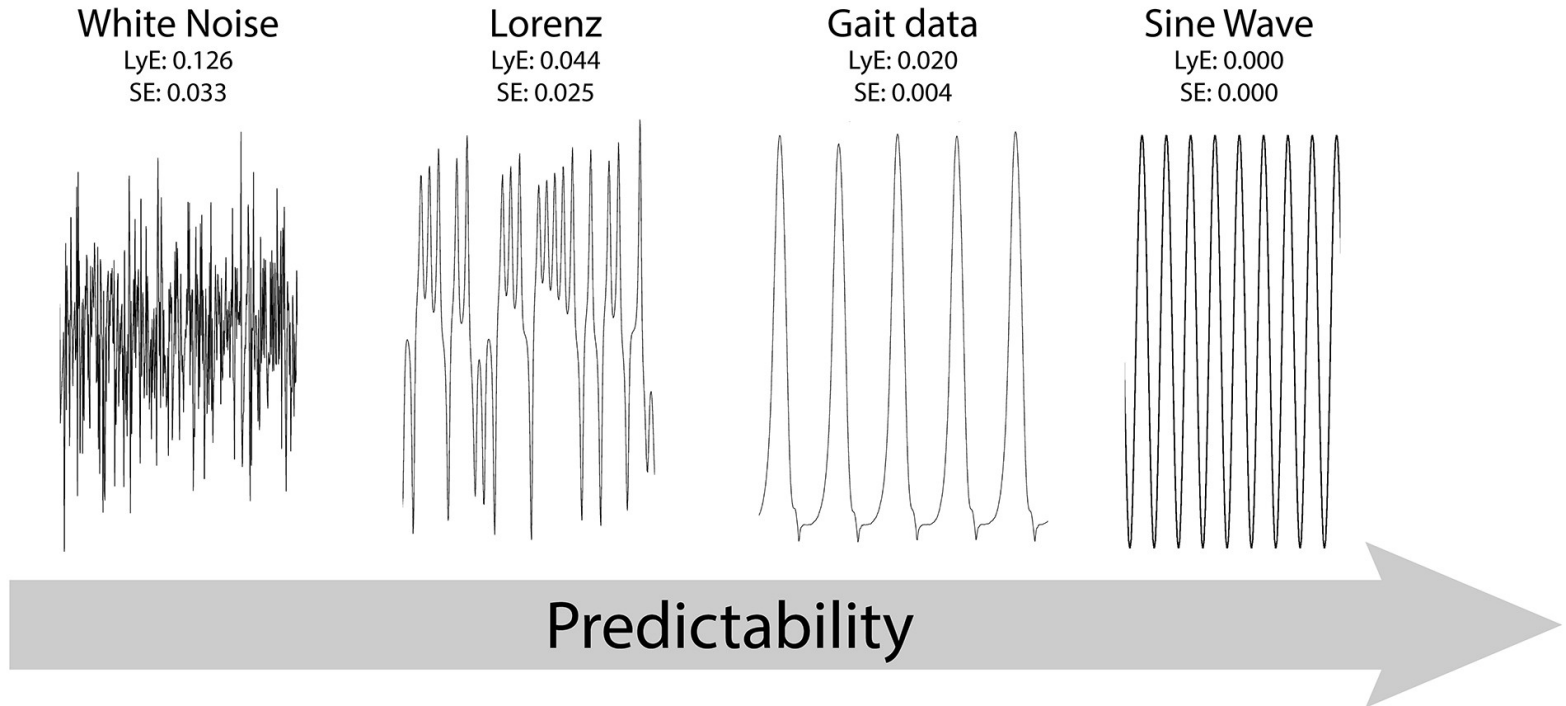
Graphical representation of three time series with known nonlinear properties and a typical gait time series. Values for LyE and SE for all for signals indicate valid representation of the predictability of a time series.

For validation purpose, both algorithms were tested on times series with known properties. As can be seen in Fig 2, both LyE and SE values gradually decrease as predictability increases, with highest values for a stochastic system such as a random white noise signal, intermediate values for a chaotic Lorenz attractor system and gait signals, and values of zero for a deterministic system such as a sine wave. All analyses were conducted in MATLAB (R2014a, MathWorks, USA). The functions and dependencies to perform concatenation are provided in Electronic Supplementary Material (ESM#1), but also updated on GitHub (https://github.com/laboratory-of-movement-biomechanics-eth/gait_concatenation).

### Statistical analysis

In order to quantify systematic (Bias) and random error (Limits of agreement—LoA) effects between full and concatenated time series, Bland-Altman (BA) analysis was performed for each of the nonlinear parameters. Additionally, intra-class correlation coefficients (ICC 3,1) and standard error of measurement (SEM) were calculated between the *fullTS* of the unperturbed walking trials (*normg*) and the concatenated time-series of the same trials to assess repeatability and measurement precision. ICC quality was interpreted according to [26]. Mixed factor ANOVAs were performed by using nonlinear exponents as dependent variables, whereas cutting conditions (*4 levels*: *fullTS*, *cut1001*, *cut0803*, *cut0605*), perturbation trials (*3 levels*: *har40*, *normg*, *wei40*) as well as the interaction of cutting conditions and perturbation trials were included as independent fixed effect variables and subjects as random effects.

In order to investigate whether the concatenation process is able to maintain known effects of perturbations to the walking patterns, paired effect sizes (ESs) were investigated on *normg* compared to *har40 and wei40*. In these exemplary trials, *fullTS* for each perturbation trial represented the ground-truth and was compared against all cutting conditions (*cut1001*, *cut0803*, *cut0605)*, where:

$$d=\frac{{\overset{-}{x}}_{1}-{\overset{-}{x}}_{2}}{s}$$ (4)

where ${\overset{-}{x}}_{1}$ corresponds to the mean of nonlinear measures of *normg* time series, ${\overset{-}{x}}_{2}$ corresponds to nonlinear measures of the perturbed time series, and *s* is defined as the pooled standard deviation of both time series. In order to assess sensitivity of the three nonlinear measures to data concatenation, the change in ESs were evaluated using coefficient of variation (CoV) of ES. All statistical analyses were performed in R (v3.4.1, The R Foundation for Statistical Computing, Austria).

## Results

The average number (±SD) of cuts differed between each concatenated time series, with *cut0605* including 23±1, *cut0803*: 17±1, and *cut1001*: 13±1 concatenations respectively. FNN and AMI resulted in an average number of embedded dimensions dim = 8.2±0.6 and an average time lag of τ = 40.3±4.4 frames. Due to non-physiological changes in the subjects’ walking patterns (missing HS due to bodyweight unloading) during the *har40*, 3 trials were removed from the analysis.

### Effects of concatenation on largest lyapunov exponent

**Fig 3:**
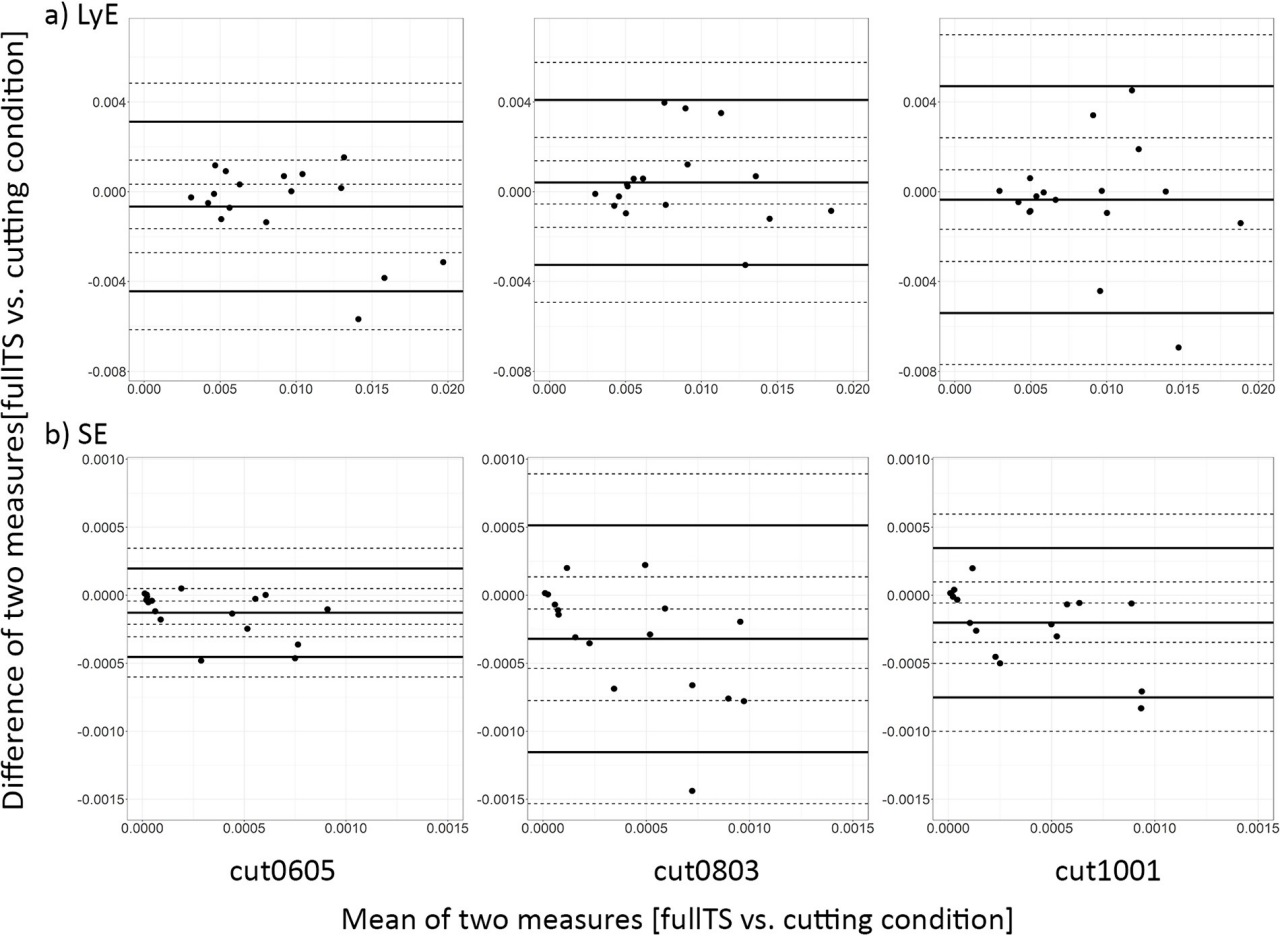
Bland-Altman plots for *fullTS* versus different cutting conditions for the perturbation trial *normg* for both measures LyE (top) and SE (bottom). The middle bold line indicates systematic bias, whereas the top and bottom bold lines indicate the upper and lower limits of agreement (LoA). Dashed lines represent 95% confidence intervals for the three estimates of bias and upper and lower LoA.

When comparing LyE for the *fullTS* against concatenated trials, ICC exhibited good to excellent results, while relative SEM showed errors of up to 20% (Table 1). Bland-Altman plots revealed good agreement for *cut0803* and *cut0605* conditions in comparison to *cut1001* (Fig 3 top). Mixed factor ANOVA revealed significant effects of perturbation trials (p<0.01) on LyE: however, there were no significant effect of concatenation conditions (p = 0.830) and no interactive effect of perturbation x concatenation conditions on LyE (p = 0.992).

**Table 1:** Reliability and precision measures for the two nonlinear methods and cutting conditions for *normg* trials.

	Method/Cut	Mean ± SD	ICC (3,1)	BIAS ± LoA	SEM	Relative SEM
**LyE**	fullTS	0.86 ± 0.44 ×10^−2^				
	cut1001	0.90 ± 0.48 ×10^−2^	0.84	-0.04 ± 0.51 ×10^−2^	0.18 ×10^−2^	0.20
	cut0803	0.82 ± 0.46 ×10^−2^	0.91	0.04 ± 0.37 ×10^−2^	0.13 ×10^−2^	0.16
	cut0605	0.93 ± 0.54 ×10^−2^	0.92	-0.07 ± 0.38 ×10^−2^	0.13 ×10^−2^	0.14
**SE**	fullTS	0.02 ± 0.03 ×10^−2^				
	cut1001	0.05 ± 0.04 ×10^−2^	0.71	-0.02 ± 0.05 ×10^−2^	0.02 ×10^−2^	0.40
	cut0803	0.06 ± 0.05 ×10^−2^	0.46	-0.03 ± 0.08 ×10^−2^	0.03 ×10^−2^	0.50
	cut0605	0.04 ± 0.04 ×10^−2^	0.87	-0.01 ± 0.03 ×10^−2^	0.01 ×10^−2^	0.25

**Fig 4:**
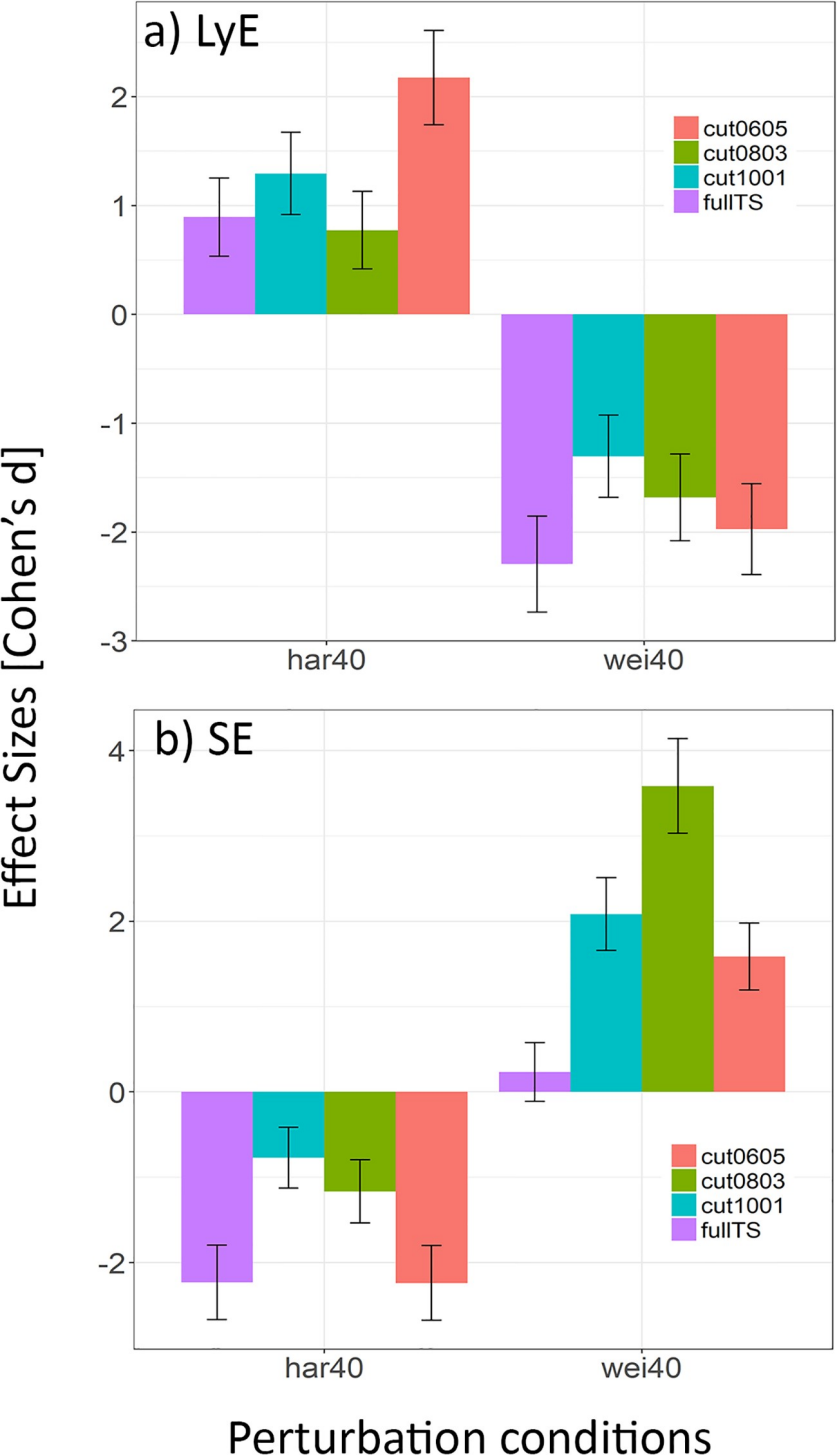
Effect sizes between *normg* trials and the two (loaded and unloaded) perturbation conditions. The uncut complete time series (fullTS) represents the true effect size resulting from the perturbation, and are compared against the effect sizes resulting from the concatenated time series for each of the nonlinear measures.

For the *fullTS*, unloading bodyweight led to positive ESs in LyE, whereas addition of weight resulted in negative ESs (Fig 4). Absolute ES magnitudes did not agree between *fullTS* and concatenated trials: in particular, *cut0605* exhibited considerably larger effects in the *har40* condition and similarly *cut1001* in the *wei40* condition. In addition, LyE showed an average intra-perturbation effect size variance of CoV = 36.3±18.4%.

### Effects of concatenation on sample entropy

ICC calculation of SE showed only fair to good results for all cutting conditions except *cut0803*. Bland-Altman plots revealed widest LoA in the *cut0803* condition and narrowest LoA in *cut0605* followed by *cut1001* (Fig 3 bottom). The relative SEM showed errors up to 50%. Mixed factor ANOVA revealed a significant effect of perturbation trials (p<0.001) on SE, but not of concatenation conditions (p = 0.191) and no significant interaction effect of perturbation x concatenation conditions (p = 0.714). Average intra-perturbation effect size variance was CoV = 60.3±19.2%.

**Fig 5:**
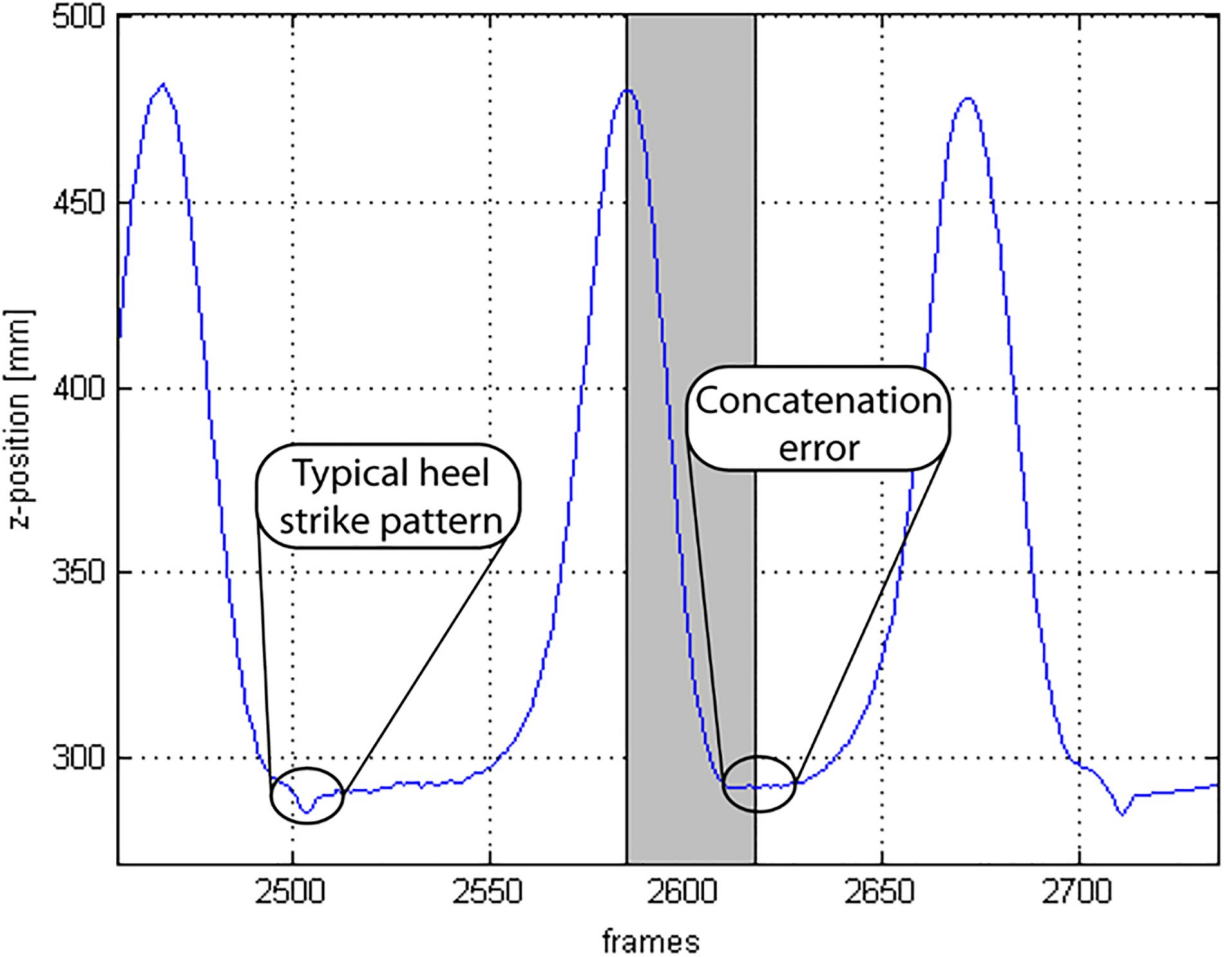
Example of possible errors resulting from the concatenation process. The grey shaded area represents transition frames.

Upon visual inspection, concatenation errors were generally only apparent in small translational perturbations in the time series such as during heel strike (Fig 5).

## Discussion

A classic problem in gait analysis is the assessment of functional characteristics requiring continuous movement data, which is often perturbed by subjects leaving the field of measurement, or by treadmills governing temporal and spatial characteristics in an artificial manner. In this study, we present a novel approach for concatenation of discontinuous time-series in order to generate a single large continuous series, hence allowing assessment of dynamic behaviour in human locomotion. In order to assess whether concatenated trials maintain both the magnitude and the direction of perturbation effects (using ESs), normal walking within this study was compared to a) walking with a harness with bodyweight support and b) with additional weight, both known to perturb gait kinematics [23, 27]. Through validation against exemplary weight induced perturbations, we have been able to demonstrate that our approach is able to concatenate multiple short time series without inducing any changes in the direction of the effect for both LyE and SE. However, concatenation resulted in considerable change in the magnitude of ES and a systematic error of up to 20% for LyE and 50% for SE. Thus the outcome of the proposed concatenation approach was not so robust for SE with a maximum systematic error equal to half the measurement value. The interpretation of regularity and stability as assessed using the proposed SE algorithm and absolute magnitude of LyE respectively should be interpreted with caution (due to their sensitivity to the size as well as pre-processing of the dataset). To overcome this issue, our method provides an approach for estimating the relative change in both regularity (via SE) and dynamic stability across perturbations or participant groups (via LyE) for both uninterrupted and interrupted time series.

LyE estimates from entire 5 min walking trials (*fullTS*) showed an average value of 0.86 ± 0.44 ×10^−2^ across all participants. This value is considerably lower than other treadmill studies evaluating foot positions, with values ranging from 0.3–0.9 [28], suggesting a much more stable walking pattern in the present study. However, comparison of nonlinear measures across studies is generally difficult, due to its known sensitivity to factors such as data length, sampling frequency and filtering [29]. For example, the FNN method identified on average 8 embedded dimensions from the foot trajectory position data of this study, which is considerably higher than the typically observed number of 5 dimensions for studies investigating accelerations of trunk kinematics [29]. In this situation the foot always returns to the floor during the stance phase of gait, thereby repetitively converging to a fixed vertical coordinate, and which might therefore explain the more stable pattern as can easily be visualized in Fig 2. Together with the extended length of our time series (<30’000 time frames per trial), those differences in considering vertical foot kinematic trajectory might well have led to the differences in the number of embedding dimensions [29].

Our concatenation approach of combining several short time series, led on average to a more reliable estimate (average ICC = 0.89) for LyE compared to ICC = 0.60 reported elsewhere [30]. However, when considering the effects of the perturbations, heterogeneity between concatenation conditions was observed. Finally, one of the pre-requisites for evaluating LyE across all conditions was to achieve identical length for each time series. In order to achieve this prerequisite for different concatenation conditions a distinct number of short time series were needed, resulting in a larger number of concatenations for certain time series (e.g. *cut0605 vs*. *cut1001*). As each concatenation is potentially associated with small errors, it could be that the reduced reliability of LyE with addition of discontinuities could also be simply due to the number of cuts but there was no significant effect of perturbation condition. Another consideration is the applied algorithm to determine LyE. In this study, Wolf’s method was used, which is slightly more accurate under only chaotic conditions as compared to Rosenstein’s method. The Rosenstein algorithm is thought to be advantageous when signals are comprised of more periodic than chaotic dynamics [31]. It appears, the characteristics of the heel trajectory time series lies between that of a chaotic and a periodic system (Fig 2). Therefore, Rosenstein’s algorithm is likely to provide more accurate estimates of LyE in comparison to that of Wolf’s. On the other hand, a recent comparison of the two algorithms finds that both algorithms are similarly efficient, when human walking and running kinematics are analysed [32]. In this respect, further work is required to evaluate these relationships, but also possibly introduce further improvements to the presented concatenation approach.

Analysis of 5 minutes of continuous treadmill walking resulted in an average SE of 0.02 ± 0.03 ×10^−2^ across all participants. Other studies assessing the regularity of times series during treadmill walking report values of SE = 0.22–0.31 [23]. Higher values of SE reflecting a more irregular / unpredictable time series [11]. Therefore, the values estimated within our study suggest comparatively more regular walking behaviour, which again is most likely caused by the choice of time series [33]. Decker and colleagues report on joint angle time series as compared to vertical marker trajectory in our study [23]. The mere difference is that the here used vertical marker trajectory is bound to always identical (near identical, when considering measurement noise) point in space (i.e. floor), which is not the case in joint angle data. Our results show only fair to good reliability between the fullTS and the concatenated time series and thus, resulting large SEM values. But the direction of the harness and weight effect did not change direction for the cut conditions. We conclude that, concatenated time series could lead to a false interpretation of the absolute SE results, and further options to improve such approaches for valid SE estimation should therefore be investigated.

In the present study, we used the heel marker for the assessment of dynamic gait behaviour. The choice of marker was based on the fact that a) in the clinical context the most commonly observed outcomes are computed from footfall kinematics [34], and b) that during walking corrections to the base of support relative to the center of mass are provided by suitably adjusting the placement of the feet [35]. The vertical trajectory of the foot was observed in order to avoid the constraints imposed by the treadmill (however, due to the fact that the concatenation is performed in three-dimensional space, the proposed procedure would also allow to investigate medio-lateral foot placement). As the trajectory of the foot in the anterior-posterior direction is likely dragged by the treadmill such that the stance foot is helped backwards. Furthermore, it was possible to evaluate the position data of the foot rather than the velocity profile, as we are fairly certain that the dataset was devoid of any non-stationarities. Finally, in order to demonstrate the effectiveness our concatenation algorithm we have evaluated the dynamic stability of the velocity of the heel marker from the concatenated trials. It is to be noted that similar to position data our approach once again revealed on average good realibility (average ICC = 0.87) for the velocity time series. Please refer to ESM#2.

For the process of concatenating, the highest priority was to retain as much data as possible. This was enforced by identifying and concatenating at the first local minima of the distance function $D(C_{1}^{n},C_{2}^{n,v})$, which implied that the final heel strike in the previous short time series was joined to the first heel strike of the subsequent short time series. However, in situations when the current time series $C_{1}^{n}$ contained an abnormal movement pattern, finding a similar pose in the subsequent time series, $C_{2}^{n,v}$ might be problematic. Alternatively, the first heel strike in $C_{2}^{n,v}$ might not always be the best match to $C_{1}^{n}$ therefore causing irregularity in the concatenated time series, i.e. concatenation error (Fig 5). In future, unrestricted selection of concatenation windows in $C_{1}^{n}$ and $C_{2}^{n,v}$ might allow further improvements to the achieved concatenation results. Moving forward, while it is certainly possible that the proposed method is generalizable to different experimental settings, marker trajectories and planes of walking, the reliability and accuracy of concatenation addressing these aspects need to be established. Importantly, future investigations should take into account that each of these aspects could likely influence the choice of transformation $T_{j}^{v}$ chosen for concatenation.

In conclusion, our results indicate that the proposed concatenation method can reliably be used for LyE estimation and to evaluate dynamic stability of the gait pattern. However, measures of SE seem to be sensitive to the proposed concatenation procedure, and are therefore subject to false interpretation. By combining multiple short time series, our concatenation approach removes current obstacles in evaluating (e.g. nonlinear) parameters that rely on collection of large continuous time series. One key advantage of the proposed concatenation method is that previously collected data can be subsequently re-processed, thereby allowing the estimation of sophisticated nonlinear metrics in existing datasets. As a result, the ability to concatenate data from several trials improves perspectives for using overground walking protocols that are limited by the field of view, such as the 8-walk [10], and still allow a comprehensive analysis of the dynamical nature of human walking in physiological manner.

## Supporting information

S1 File The codes for the concatenation algorithm are attached as a zip file. (ZIP)

S2 File Additional observations on comparing dynamic stability for position to velocity profiles are presented. **Table A in S2 File Reliability and precision metrics for LyE derived from position and velocity of the foot in the vertical direction**. **Table A in S2 File** provides LyE for the fullTS vs. all the other concatenation conditions (cut1001, cut0803, cut0605) for both position as well as velocity. The ICC(3,1) for velocity ranged between 0.83–0.93, with lower levels of relative SEM (comparing the SEM for the concatenation to the fullTS) in comparison to the LyE for position time series.
(DOCX)

S1 Fig Comparison of position and velocity time series from the foot in the vertical direction. **S1 Fig** provides the comparison of position vs velocity timeseries in the vertical direction.
(PNG)

S2 Fig Effect sizes for the two perturbation conditions for position data of the foot in the vertical direction. **S2 Fig** shows that while the LyE for position harness led to more stable pattern, LyE for velocity resulted in less stable pattern across all concatenation conditions as well as fullTS.
(TIFF)

S3 Fig Effect sizes for the two perturbation conditions for velocity data of the foot in the vertical direction. **S3 Fig** shows that while the LyE for position harness led to more stable pattern, LyE for velocity resulted in less stable pattern across all concatenation conditions as well as fullTS.
(TIFF)
