# Supplementary material for: A method to concatenate multiple short time series for evaluating dynamic behaviour during walking
Source: PLoS One. 2019 Jun 21;14(6):e0218594. doi: 10.1371/journal.pone.0218594 (PMC6588245; doi:10.1371/journal.pone.0218594)

Marker-Set

| Name |  | Segment | Location |
| --- | --- | --- | --- |
| RTO1 |  | Foot - right | Caput metatarsale I |
| RTO3 |  | Foot - right | Metatarsale III |
| RTO5 |  | Foot - right | Caput metatarsale VI |
| RHEE |  | Foot - right | Calcaneus |
| RMMA |  | Shank - right | Medial malleolus |
| RLMA |  | Shank - right | Lateral malleolus |
| RTMT |  | Shank - right | unspecified Tibia, ventral |
| RTLF |  | Shank - right | unspecified Fibula, lateral |
| RTTT |  | Shank - right | Tuberositas tibea |
| RTIB |  | Shank - right | Caput fibulae |
| RMCO |  | Thigh - right | Medial femur epicondylus |
| RLCO |  | Thigh - right | Lateral femur epicondylus |
| RTFR |  | Thigh - right | unspecified Femur, front distal |
| RTLL |  | Thigh - right | unspecified Femur, lateral distal |
| RTLH |  | Thigh - right | unspecified Femur, lateral proximal |
| RASI |  | Pelvis | Spina illiaca ant. sup. – right |
| RTMS |  | Pelvis | Iliaca – right |
| RPSI |  | Pelvis | Spina illiaca post. Sup – right |
| SACR |  | Pelvis | Sacrum |
| LPSI |  | Pelvis | Spina illiaca ant. sup. – left |
| LTMS |  | Pelvis | Iliaca - left |
| LASI |  | Pelvis | Spina illiaca post. Sup - left |
| LTO1 |  | Foot - left | Caput metatarsale I |
| LTO3 |  | Foot - left t | Metatarsale III |
| LTO5 |  | Foot - left | Caput metatarsale VI |
| LHEE |  | Foot - left | Calcaneus |
| LMMA |  | Shank - left | Medial malleolus |
| LLMA |  | Shank - left | Lateral malleolus |
| LTMT |  | Shank - left | unspecified Tibia, ventral |
| LTLF |  | Shank - left | unspecified Fibula, lateral |
| LTTT |  | Shank - left | Tuberositas tibea |
| LTIB |  | Shank - left | Caput fibulae |
| LMCO |  | Thigh - left | Medial femur epicondylus |
| LLCO |  | Thigh - left | Lateral femur epicondylus |
| LTFR |  | Thigh - left | unspecified Femur, front distal |
| LTLL |  | Thigh - left | unspecified Femur, lateral distal |
| LTLH |  | Thigh - right | unspecified Femur, lateral proximal |
| RWRA |  | Forearm - right | Proc. styloideus radii |
| RWUL |  | Forearm - right | Proc. styloideus ulnae |
| RFRA |  | Forearm - right | unspecified forearm, radial |
| RFUL |  | Forearm - right | unspecified forearm, ulnae |
| RMEC |  | Humerus - right | Medial epicondylus |
| RLEC |  | Humerus - right | Lateral epicondylus |
| RHLT |  | Humerus - right | unspecified upperarm, lateral |
| RHVT |  | Humerus - right | unspecified upperarm, ventral |
| RSHO |  | Shoulder | Acromion - right |
| MSTC |  | Shoulder | Manubrium sterni cranial |
| CVC7 |  | Shoulder | C7 |
| LSHO |  | Shoulder | Acromion - left |
| LWRA |  | Forearm - left | Proc. styloideus radii |
| LWUL |  | Forearm - left | Proc. styloideus ulnae |
| LFRA |  | Forearm - left | unspecified forearm, radial |
| LFUL |  | Forearm - left | unspecified forearm, ulnae |
| LMEC |  | Humerus - left | Medial epicondylus |
| LLEC |  | Humerus - left | Lateral epicondylus |
| LHLT |  | Humerus - left | unspecified upperarm, lateral |
| LHVT |  | Humerus – left | unspecified upperarm, ventral |
| RFHD |  | Head | Headband |
| RBHD |  | Head | Headband |
| LFHD |  | Head | Headband |
| LBHD |  | Head | Headband |


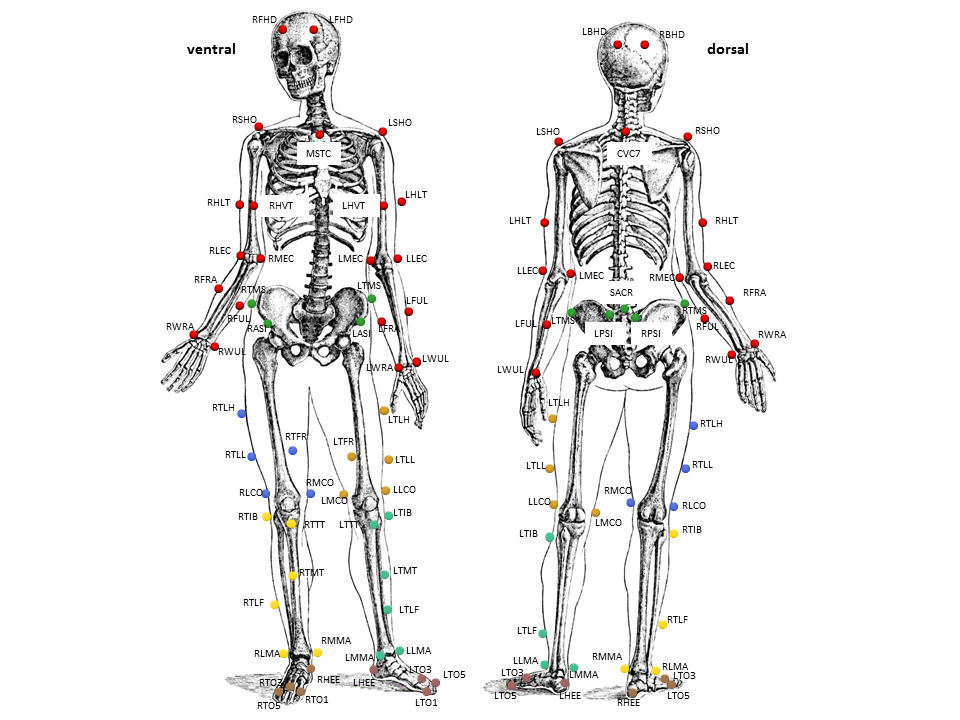

Supplement: S1 File — (ZIP) [file pone.0218594.s001.zip › Electronic Supplementary Material/ESM#1_ConcatenationKinematics_Codes/Suppl.Material.Codes/Marker-Attachments.docx]
