# Supplementary figures and images for: A method to concatenate multiple short time series for evaluating dynamic behaviour during walking
Source: PLoS One. 2019 Jun 21;14(6):e0218594. doi: 10.1371/journal.pone.0218594 (PMC6588245; doi:10.1371/journal.pone.0218594)

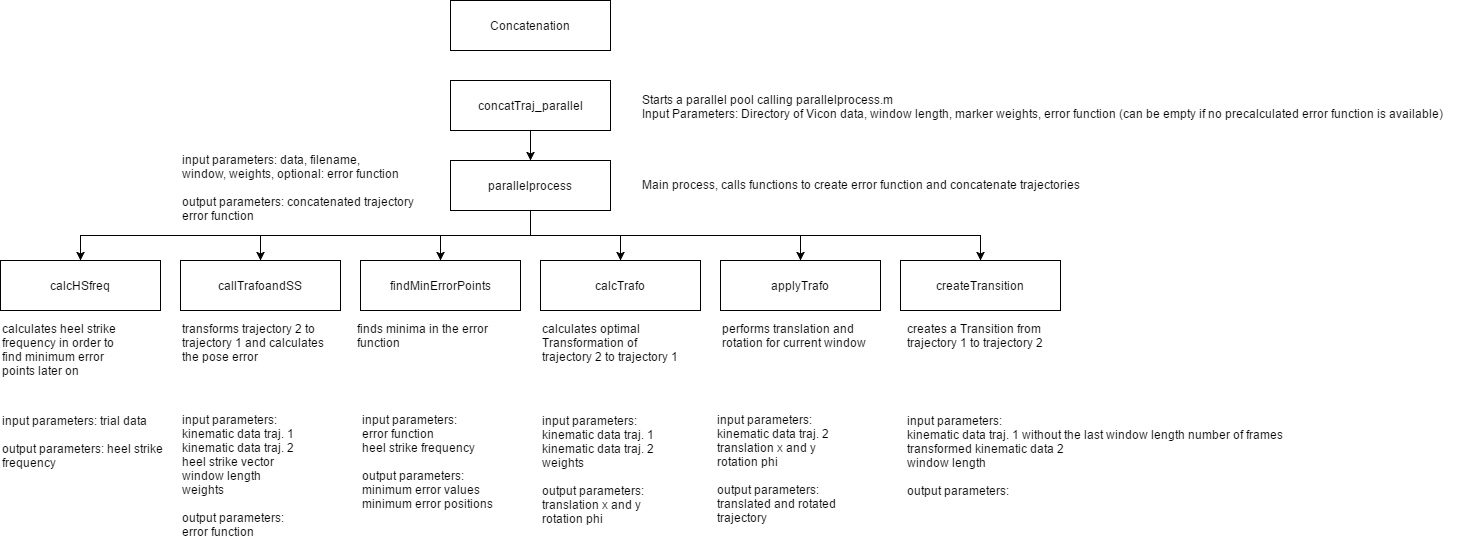

Supplement: S1 File — (ZIP) [file pone.0218594.s001.zip › Electronic Supplementary Material/ESM#1_ConcatenationKinematics_Codes/Suppl.Material.Codes/01_concatenationcodes_flow_chart.png]

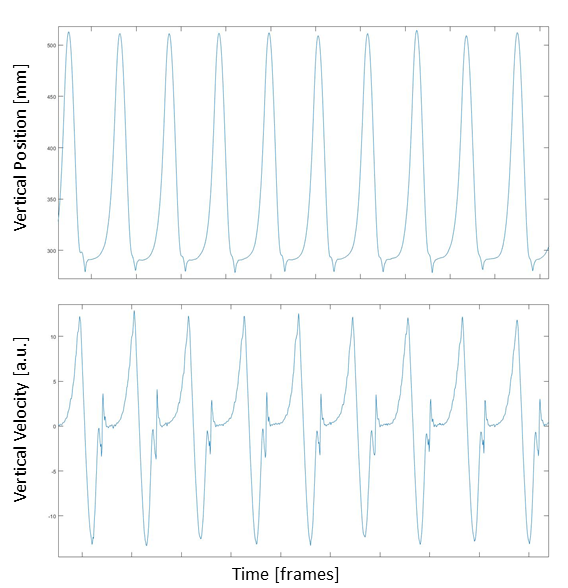

Supplement: S1 File — (ZIP) [file pone.0218594.s001.zip › Electronic Supplementary Material/ESM#2_Position_vs_Velocity/ESM_Fig 1.png]

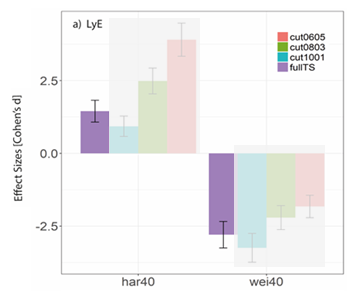

Supplement: S1 File — (ZIP) [file pone.0218594.s001.zip › Electronic Supplementary Material/ESM#2_Position_vs_Velocity/ESM_Fig 2.png]

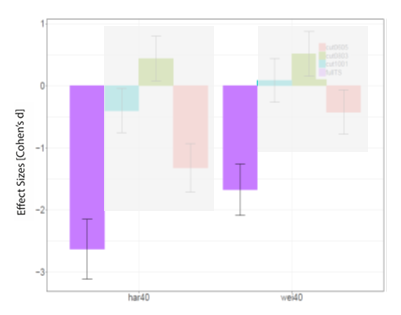

Supplement: S1 File — (ZIP) [file pone.0218594.s001.zip › Electronic Supplementary Material/ESM#2_Position_vs_Velocity/ESM_Fig 3.png]

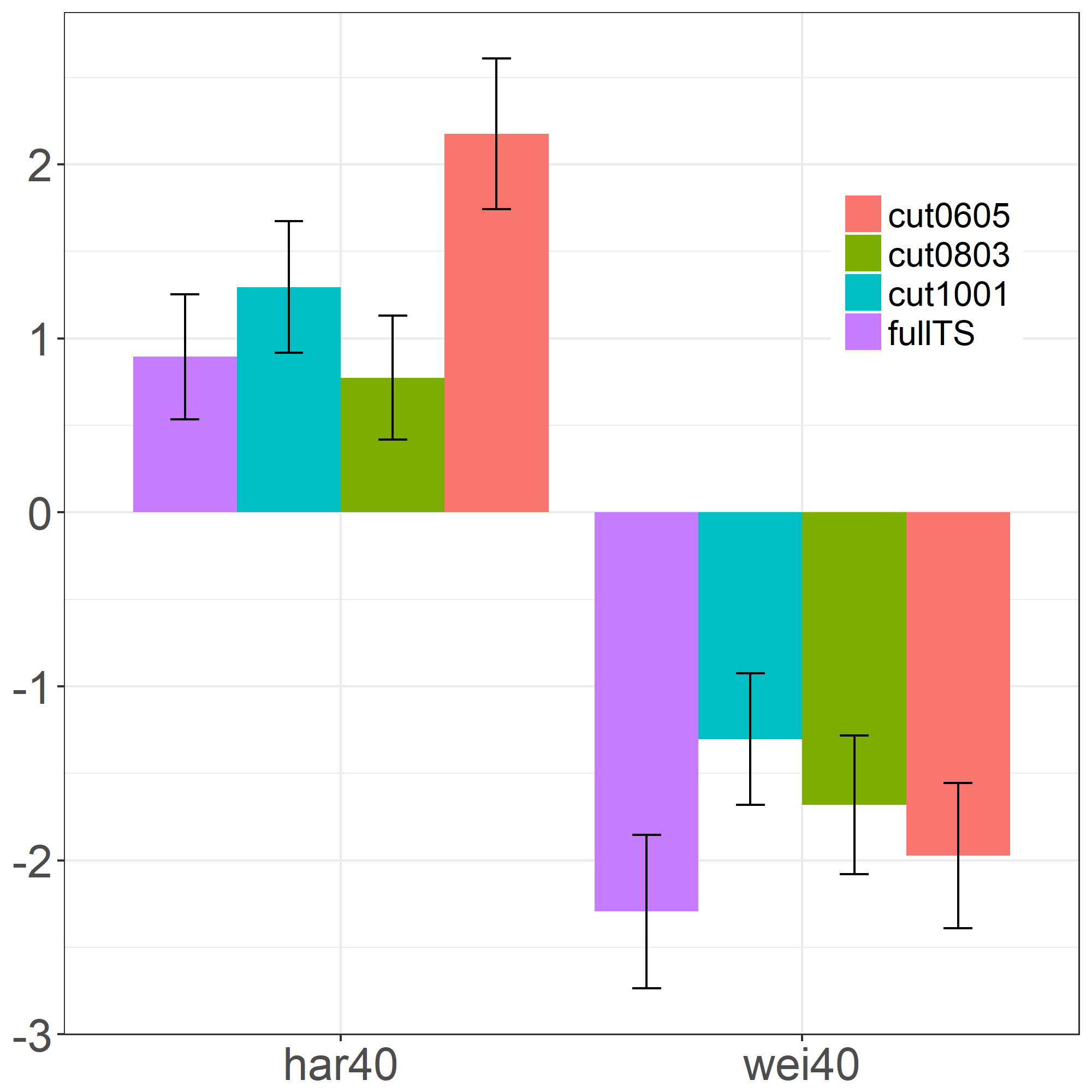

Supplement: S2 Fig — shows that while the LyE for position harness led to more stable pattern, LyE for velocity resulted in less stable pattern across all concatenation conditions as well as fullTS. (TIFF) [file pone.0218594.s004.tiff]

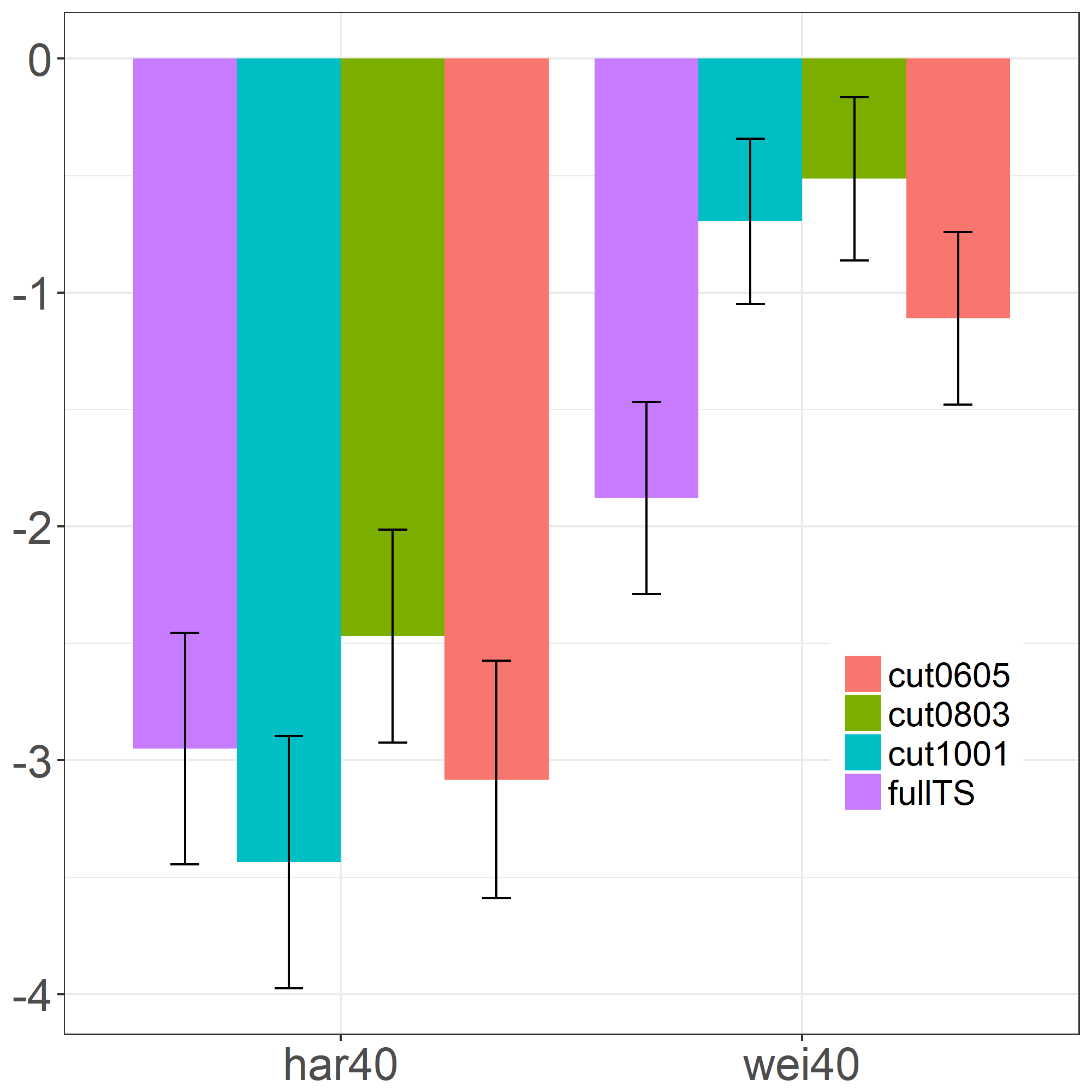

Supplement: S3 Fig — shows that while the LyE for position harness led to more stable pattern, LyE for velocity resulted in less stable pattern across all concatenation conditions as well as fullTS. (TIFF) [file pone.0218594.s005.tiff]
