# Supplementary material for: A method to concatenate multiple short time series for evaluating dynamic behaviour during walking
Source: PLoS One. 2019 Jun 21;14(6):e0218594. doi: 10.1371/journal.pone.0218594 (PMC6588245; doi:10.1371/journal.pone.0218594)
Supplement: S2 File — Table A in S2 File Reliability and precision metrics for LyE derived from position and velocity of the foot in the vertical direction. Table A in S2 File provides LyE for the fullTS vs. all the other concatenation conditions (cut1001, cut0803, cut0605) for both position as well as velocity. The ICC(3,1) for velocity ranged between 0.83–0.93, with lower levels of relative SEM (comparing the SEM for the concatenation to the fullTS) in comparison to the LyE for position time series. (DOCX) [file pone.0218594.s002.docx]

**A method to concatenate multiple short time series for evaluating dynamic behaviour during walking**

Stefan Orter^1^; Deepak K. Ravi^1^; Navrag B. Singh^1^; Florian Vogl^1^; William R. Taylor^1^; Niklas König Ignasiak^1,2^

**Electronic Supplementary Material (ESM#2)**

**Additional observations on comparing dynamic stability for position and velocity profiles of foot kinematics**

In the current study, vertical position data of the foot is observed during treadmill walking in three different conditions: normal walking, walking with a harness system and walking with additional body weight. This was done in order to assess whether concatenation of multiple time series would lead to differences in the findings on dynamic stability. For example, larger values of the computed largest Lyapunov exponent (LyE) in the perturbation condition as compared to normal walking would be interpreted as a more unstable pattern. At this point we don’t know how the perturbation would affect the stability of the pattern in the vertical direction. We have evaluated dynamic stability on the vertical position trajectory of a heel marker as well as using the velocity of the same trajectory by calculating the first derivative of the position data. The visual inspection of both signals reveal that the velocity profiles are considerably more “jerky” (S1 Fig).

**S1 Fig. Comparison of position and velocity data.**

However, despite such inconsistencies in the original signal the concatenation results in general, appear quite reliable (in fact the ICC as well as relative SEM indicate slightly better reliability in comparison to position time series – S1 Table).

**Table A. Reliability and precision metrics for LyE derived from position and velocity data**

|  | **Method/Cut** | **Mean ± SD** | **ICC (3,1)** | **BIAS ± LoA** | **SEM** | **Relative SEM** |
| --- | --- | --- | --- | --- | --- | --- |
| **Position** | fullTS | 0.86 ± 0.44 ×10^-2^ |  |  |  |  |
|  | cut1001 | 0.90 ± 0.48 ×10^-2^ | 0.84 | -0.04 ± 0.51 ×10^-2^ | 0.18 ×10^-2^ | 0.20 |
|  | cut0803 | 0.82 ± 0.46 ×10^-2^ | 0.91 | 0.04 ± 0.37 ×10^-2^ | 0.13 ×10^-2^ | 0.16 |
|  | cut0605 | 0.93 ± 0.54 ×10^-2^ | 0.92 | -0.07 ± 0.38 ×10^-2^ | 0.13 ×10^-2^ | 0.14 |
| **Velocity** | fullTS | 1.19 ± 0.39 ×10^-2^ |  |  |  |  |
|  | cut1001 | 1.12 ± 0.36 ×10^-2^ | 0.85 | 0.07 ± 0.40 ×10^-2^ | 0.14 ×10^-2^ | 0.13 |
|  | cut0803 | 1.17 ± 0.35 ×10^-2^ | 0.83 | 0.01 ± 0.41 ×10^-2^ | 0.15 ×10^-2^ | 0.13 |
|  | cut0605 | 1.21 ± 0.37 ×10^-2^ | 0.93 | -0.02 ± 0.28 ×10^-2^ | 0.10 ×10^-2^ | 0.08 |

In the following we will focus on the effect of the **perturbation condition** (harness and weight vest) compared to the walking behavior during normal walking on the treadmill. The effect is displayed as effect size (Cohen’s d), which is calculated according to the following equation:

$$d=\frac{normal gait-perturbed gait}{pooled standard deviation}$$

Hence, positive effect sizes indicate a more stable pattern in the perturbed condition (smaller LyE values indicate higher stability) and negative effect sizes would be interpreted as more stability in the normal gait conditions. For this consideration we want to ignore possible effects caused by our concatenation approach but instead focus on the un-concatenated full time series (fullTS; *true data*). The following plot provides the identified effect sizes for the perturbation conditions found in the position data. As can be seen, the harness condition resulted in a more stable pattern, whereas the weight vest caused the dynamics to be less stable (S2 Fig).

**S2 Fig. Effect sizes for the two perturbation conditions for position data.**

For the velocity profiles, it turns out that both perturbation conditions - harness as well as weight vest conditions - result in a less stable pattern (S3 Fig). It has been claimed that position and velocity profiles describe different aspects of the system and therefore cannot be directly compared (Mehdizadeh 2018: *The largest Lyapunov exponent of gait in young and elderly individuals: A systematic review* *[29]*). Our observation confirms this statement. We believe that position data is related to the spatial organisation of movement behaviour, whereas velocity data informs about the spatio-temporal dynamics during walking. Hence, we caution to transfer position data into velocities as a strategy to manage non-stationarity in time series, because this might change the object under investigation.

**S3 Fig. Effect sizes for the two perturbation conditions for velocity data.**

Furthermore, this observation has interesting implications for the non-linear observations of human movement behavior. Typically, the largest Lyapunov exponent is used to investigate the dynamic stability in a system across perturbations or cohorts. Importantly, the dynamics of the system are unknown before the observation and are only interpreted afterwards relative to each other. Furthermore, there seems to be no general consensus what form of kinematic signal (position, velocity or acceleration data) should be analyzed to quantify the stability of the system. However, our data confirms that position, velocity and potentially acceleration signals encode entirely different aspect of the dynamic behavior. For the clinical application of those methods this relates to several such questions which should be further investigated.

In the current situation, we feel it is important that researchers validate their non-linear analysis method based on signals with known dynamics (random, chaotic, periodic) and to interpret the results within this internal validation. However, because of the sensitivity of non-linear analysis methods to aspects such as data type, data length and filtering it currently seems difficult to summarize results across multiple studies and to derive more generalizable inferences about human gait dynamics control.
